# Age Impacts the Burden That Reference Memory Imparts on an Increasing Working Memory Load and Modifies Relationships With Cholinergic Activity

**DOI:** 10.3389/fnbeh.2021.610078

**Published:** 2021-02-10

**Authors:** Victoria E. Bernaud, Ryoko Hiroi, Mallori L. Poisson, Arthur J. Castaneda, Ziv Z. Kirshner, Robert B. Gibbs, Heather A. Bimonte-Nelson

**Affiliations:** ^1^Department of Psychology, Arizona State University, Tempe, AZ, United States; ^2^Arizona Alzheimer's Consortium, Phoenix, AZ, United States; ^3^Department of Pharmaceutical Sciences, University of Pittsburgh School of Pharmacy, Pittsburgh, PA, United States

**Keywords:** female, rat, attention, cholinergic system, reference memory, working memory, water radial-arm maze, aging

## Abstract

Rodent aging research often utilizes spatial mazes, such as the water radial-arm-maze (WRAM), to evaluate cognition. The WRAM can simultaneously measure spatial working and reference memory, wherein these two memory types are often represented as orthogonal. There is evidence, however, that these two memory forms yield interference at a high working memory load. The current study systematically evaluated whether the presence of a reference memory component impacts handling of an increasing working memory load. Young and aged female rats were tested to assess whether aging impacts this relationship. Cholinergic projections from the basal forebrain to the hippocampus and cortex can affect cognitive outcomes, and are negatively impacted by aging. To evaluate whether age-related changes in working and reference memory profiles are associated with cholinergic functioning, we assessed choline acetyltransferase activity in these behaviorally-tested rats. Results showed that young rats outperformed aged rats on a task testing solely working memory. The addition of a reference memory component deteriorated the ability to handle an increasing working memory load, such that young rats performed similar to their aged counterparts. Aged rats also had challenges when reference memory was present, but in a different context. Specifically, aged rats had difficulty remembering which reference memory arms they had entered within a session, compared to young rats. Further, aged rats that excelled in reference memory also excelled in working memory when working memory demand was high, a relationship not seen in young rats. Relationships between cholinergic activity and maze performance differed by age in direction and brain region, reflecting the complex role that the cholinergic system plays in memory and attentional processes across the female lifespan. Overall, the addition of a reference memory requirement detrimentally impacted the ability to handle working memory information across young and aged timepoints, especially when the working memory challenge was high; these age-related deficits manifested differently with the addition of a reference memory component. This interplay between working and reference memory provides insight into the multiple domains necessary to solve complex cognitive tasks, potentially improving the understanding of complexities of age- and disease- related memory failures and optimizing their respective treatments.

## Introduction

The field of learning and memory has historically utilized a variety of methods to assess spatial navigation during learning and memory tasks, ranging from Tolman's sunburst maze to test rodents (Tolman, 1948) to the virtual reality techniques used in more recent human neuroimaging studies (Spiers et al., 2001; see review: Burgess et al., 2002). There is a long history of work showing memory decline during normal aging in humans (Salthouse et al., 1989; Cherry et al., 1993; Weaver Cargin et al., 2007; see reviews: Glisky, 2007; Harada et al., 2013; Cohen et al., 2019) and in rodent models (Barnes, 1979; Frick et al., 1995; Bimonte et al., 2003; Bizon et al., 2009; see reviews: Gallagher and Nicolle, 1993; Rodefer and Baxter, 2007; Foster, 2012; Bettio et al., 2017). Mazes requiring rodents to navigate through space to successfully solve a cognitive task have been used for decades (Bimonte-Nelson, 2015a), often focusing on hippocampal-related spatial learning and memory, where performance has consistently been shown to decline with age (Schimanski and Barnes, 2015). There is a rich history of theory and operational definitions conceptualizing the way that maze tasks are learned and solved, which has informed and driven much of the learning and memory literature (Baddeley and Hitch, 1974; Brown et al., 1993; for reviews: O'Keefe and Nadel, 1978; Bimonte-Nelson, 2015a). Spatial learning and memory in rodents can be categorized into multiple domains, most commonly spatial working memory, which is a form of short-term memory that must be constantly updated, and spatial reference memory, which is a form of long-term memory that remains fixed across time (Frick et al., 1995; Bizon et al., 2009; Foster, 2012; Bimonte-Nelson et al., 2015; for origin of definitions see: Olton, 1979; Olton and Papas, 1979; Jarrard et al., 1984). In the preclinical literature, working, and reference memory are often described as representing two separate memory systems, and are quantified as such (Olton, 1979; Jarrard et al., 1984, 2012; Dennes and Barnes, 1993; Bimonte-Nelson et al., 2003, 2015; Braden et al., 2011; Camp et al., 2012; Auger and Floresco, 2014; Bimonte-Nelson, 2015c; Hiroi et al., 2016).

Prominent in the rodent literature is the use of the radial-arm maze (RAM) (Olton and Samuelson, 1976; Ward et al., 1999; Bimonte-Nelson et al., 2004; Braden et al., 2011; Prakapenka et al., 2018; Koebele et al., 2019; see reviews: Olton, 1979; Bimonte-Nelson et al., 2010, 2015), a multi-arm apparatus requiring rodents to locate a reinforcer at the ends of particular arms, often by navigating through space using distinctive extramaze cues for optimal performance. The RAM is a win-shift task, with optimal performance involving the animal receiving a reinforcer in one location and then “shifting” away to a new location for the next reinforcer, rather than returning to the initial site of reinforcement within a testing session; this is accomplished by presenting reinforcement only upon first entry into a given arm (Bimonte-Nelson et al., 2015). In this manner, the RAM evaluates working memory, as an animal must update where it has already visited within a session. As reinforcers are removed and trials progress within a day, more items need to be remembered and the working memory load increases. The RAM can test working memory alone by providing a reinforcement in every arm, or the protocol can be modified to simultaneously test working memory and reference memory (Bimonte-Nelson et al., 2015). When evaluating working and reference memory simultaneously on the RAM, only a specific subset of the arms is associated with reinforcement; in this manner, the arms that contain a reinforcer become working memory arms, and arms that never contain a reinforcer become reference memory arms (Bimonte-Nelson et al., 2015). The capability of the RAM to efficiently and simultaneously assess working memory and reference memory, particularly when there are concerns for prior cognitive testing effects, generalization, or satiation with an extended behavioral battery (Blokland and Raaijmakers, 1993; Serrano Sponton et al., 2018), makes the RAM especially useful when evaluating variables likely to be impacted by these concerns. On the appetitively-motivated land version of the RAM, performance in aged rats has been shown to be compromised compared to young rats (Luine and Hearns, 1990; Luine and Rodriguez, 1994; Ward et al., 1999; Bimonte-Nelson et al., 2015).

Our laboratory has shown that rodents display age-related deficits in both working and reference memory domains on the water radial-arm maze (WRAM), a water-escape version of the RAM; this has been demonstrated on the 12-arm (Bimonte et al., 2003), and the 8-arm (Bimonte-Nelson et al., 2003, 2004; Koebele et al., 2017) WRAM tasks. While we have shown age-related deficits on WRAM tasks with both working and reference memory components, even when the number of working memory arms and the working memory load differed between the 12-arm and 8-arm mazes, it has not been determined whether the presence of reference memory arms impacted these outcomes. Deciphering whether age impacts the ability to handle an increasing working memory load when reference memory arms are not present is critical to better understand the parameters of age-associated memory changes.

Using the WRAM, we have found that errors made into working and reference memory arms typically occur in concert, indicating that working and reference memory demands impact each other (Braden et al., 2011, 2017; Mennenga et al., 2015b,c; Prakapenka et al., 2018; Koebele et al., 2019). Here, we evaluate relationships between working and reference memory by systematically testing working memory performance with and without the presence of reference memory demands. We test whether the ability to handle an increasing working memory load is impacted by the simultaneous necessity to handle reference memory information, as well as whether successful learning and maintenance of reference memory is affected by an increasing working memory load. Since we aim to better understand these relationships with aging, both young and aged rats are included in the current experiment.

While decades of spatial navigation work has demonstrated that aging impacts both spatial working memory outcomes (Bimonte et al., 2003; Coppola et al., 2014; Uresti-Cabrera et al., 2015) and spatial reference memory outcomes (Gage et al., 1984; Frick et al., 1995), much of this early work was done with males. More recent work testing both males and females during aging underscores the importance of characterizing unique cognitive outcomes in females (Bimonte et al., 2000; Bowman, 2005; Benice et al., 2006; Talboom et al., 2014). This is especially poignant given the recent position statements from the National Institutes of Health to include females in clinical and preclinical research (Clayton and Collins, 2014; Miller et al., 2017), and with the burgeoning research area of menopause and hormone therapies as aging ensues (Luine and Rodriguez, 1994; Gibbs, 2003; Lewis et al., 2008; Braden et al., 2011; Koebele et al., 2017). Therefore, we aim to characterize how aging in females impacts the relationship between spatial working and reference memory.

Cholinergic projections from the basal forebrain to the hippocampus and cortex play important roles in spatial learning and memory, in part *via* effects on stimulus detection and attention (Everitt and Robbins, 1997; Gibbs, 2010; Koebele and Bimonte-Nelson, 2017; Parikh and Bangasser, 2020; Venkatesan et al., 2020). These projections are negatively affected by normal aging and with neurodegenerative diseases such as Alzheimer's disease and Parkinson's disease (Drachman et al., 1980; Mesulam et al., 1987; Beach et al., 2000; Mufson et al., 2002; Gibbs, 2003; Schliebs and Arendt, 2011). Here, in behaviorally tested females, we evaluate choline acetyltransferase (ChAT) activity in the hippocampus and frontal cortex as an indicator of cholinergic function. This experimental design provides the unique opportunity to further explore how age and differential memory demands modulate the relationship between cholinergic activity and cognitive performance. Collectively, this work characterizes relationships between spatial reference and working memory outcomes in young and aged female rats, and explores whether ChAT activity in specific brain regions varies in relationship to memory depending on task demands at these two age timepoints. We hypothesize that the addition of reference memory will detrimentally impact working memory trajectory as the load increases, which will exhibit a greater burden with age. Furthermore, we hypothesize that these outcomes will correlate with cholinergic findings in regions critical to spatial learning and memory.

## Methods

### Animals

Twenty young (4 months of age) and 19 aged (21 months of age) female Fischer-344 rats were ordered at the same time from the National Institute on Aging colony at Charles River Laboratories in Raleigh, North Carolina. All animals arrived in the same shipment, were pair-housed upon arrival, and were maintained together in the same room, which was set on a 12-h light/dark cycle (lights on at 7:00 a.m.). Rats were given food and water freely throughout the study. All experimental procedures were approved by the Institutional Animal Care and Use Committee at Arizona State University and adhered to the standards of the National Institutes of Health.

### Timeline

**Figure 1:**
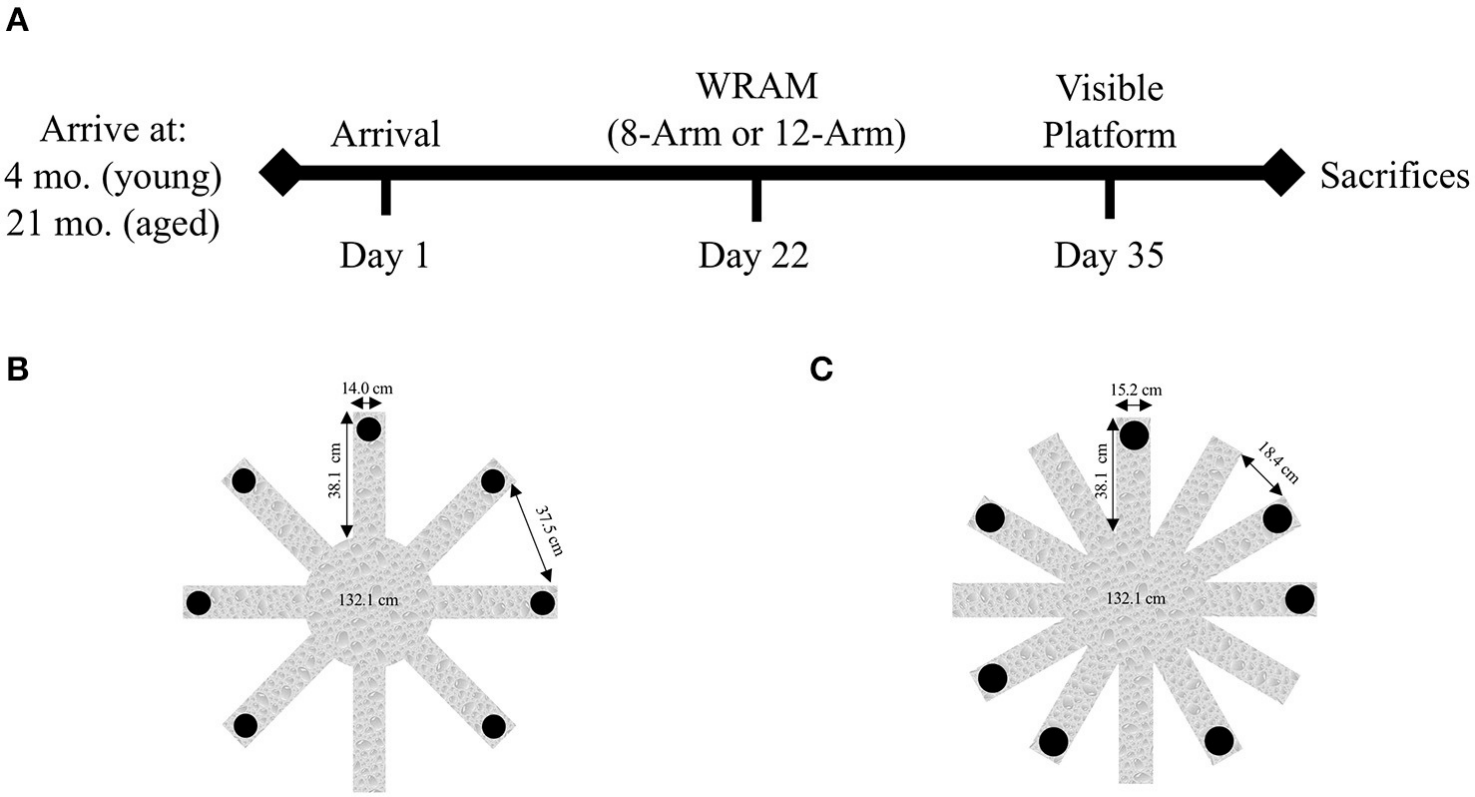
Study timeline and WRAM configurations. **(A)** The study timeline depicts the age at arrival and the commencement of behavioral testing, including WRAM and VP. **(B)** The 8-Arm WRAM apparatus, with seven of the eight arms platformed. Besides the starting arm, all other arms in the 8-Arm WRAM are spatial working memory arms. **(C)** The 12-Arm WRAM apparatus, with seven of the 12 arms platformed, making for a total of seven working memory arms, like that of the 8-Arm WRAM. This maze, however, has five reference memory arms, allowing for the analysis of both spatial reference and working memory performance on the WRAM.

The experimental timeline is depicted in Figure 1A. Behavioral testing was run on the same timeline for all subjects, beginning 21 days after their arrival. Ten young rats and nine aged rats were assigned to the 8-Arm WRAM (Figure 1B), and separate groups of 10 young rats and 10 aged rats were assigned to the 12-Arm WRAM (Figure 1C). Behavioral testing began each morning at the same time of day, and the 8-Arm WRAM was in a room separate from the 12-Arm WRAM. Animals were removed from their pair-housed cage in the vivarium, and placed individually into their own separate testing cage. Upon completion of testing for that day, each animal was returned to its pair-housed cage in the vivarium. Testing order was counter-balanced by age within each testing room to control for age-related effects due to testing order. After WRAM testing, rats were evaluated for their ability to perform the procedural components of a water-escape task using the 1-day Visible Platform (VP) task. Rats were euthanized and the brains dissected the day following completion of the behavioral battery.

### Eight-Arm Water Radial Arm Maze

The 8-Arm WRAM was used to test working memory as working memory load increased, as described previously (Bimonte-Nelson, 2015d). Figure 1B contains a schematic of the 8-Arm maze configuration. Escape platforms were hidden ~3 cm under the surface of the water in seven of the eight arms, and the water was made opaque with the addition of non-toxic black paint. The temperature of the water was kept at a range of 18–20°C. Salient external cues were placed in the testing room, including two panels on the walls outside the maze, the door, a row of tables with heating lamps, and the experimenter at the starting arm.

The subject was released into the start arm and given 3 min to find a platform. If the rat did not find a platform in the allotted trial time of 3 min it was guided to the nearest platform. Once on the platform, the rat remained for a total for 15 s before returning to the warmed home cage for a 30 s inter-trial interval. During this time, the platform recently found was removed, and the maze was cleaned of debris and water was swept over to eliminate any possible odor cues. The animal was then brought back to the start arm, where it was allowed to find another platform. This continued until all seven platforms were located; in this way, the working memory load was increased as each trial progressed, since rats needed to remember not to return to arms found previously that day. Once all seven platforms were located, the rat was returned to its heated home cage and testing began for the next animal. Testing continued in this fashion for a total of 12 days.

**Figure 2:**
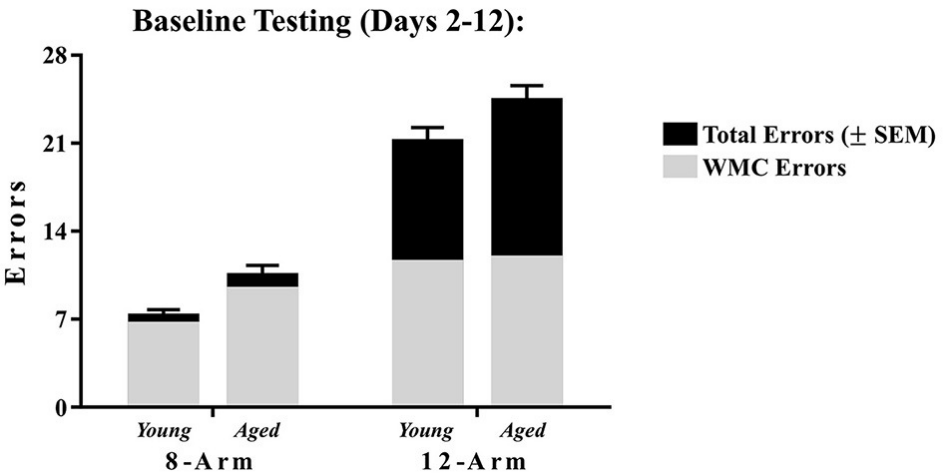
WRAM performance represented as WMC errors and their contribution to Total errors. The proportion of Total errors (±SEM) comprised of WMC errors for both mazes across Acquisition and Asymptotic Phases of testing. For the 8-Arm WRAM, other than start arm errors, all errors must be WMC errors; however, in the 12-Arm WRAM, rats can make WMC errors, as well as RM and WMI errors. While the rats tested on the 8-Arm WRAM rarely made entries into the only arm without a platform i.e., the start arm, rats tested on the 12-Arm WRAM made a large number of unplatformed arm entries. This further demonstrates the separation of errors made when a reference memory component is added to the working memory portion of the WRAM.

Arm entries were counted when the tip of the rat's snout passed 11 cm into each arm, marked by a line on the outside of the maze arms. Measures of working memory errors were quantified as done for previous research using the WRAM (Braden et al., 2010; Bimonte-Nelson, 2015d; Prakapenka et al., 2018). Working Memory Correct (WMC) errors were the number of entries into an arm that previously contained a platform for that session. Start Arm (START) errors were the number of entries into the start arm; as expected, animals made few START errors on the WRAM task, and therefore analyses of START errors were not included in subsequent analyses for the 8-Arm WRAM. The sum of all errors made for each trial was defined as Total Memory (Total) errors. Of note, for this 8-Arm maze, Total errors were nearly identical to that of WMC errors across testing days due to the low number of start arm entries, as schematically depicted in Figure 2. Errors were evaluated for each daily session and were analyzed across all days of testing.

### Twelve-Arm Water Radial Arm Maze

To determine the impact of the addition of reference memory on working memory performance as working memory load increases, the 12-Arm WRAM was used as described previously (Bimonte et al., 2003). Figure 1C contains a schematic of the 12-Arm maze. Platforms were hidden about 3 cm under the surface of the water, which was made opaque with non-toxic black paint, at seven of the 12 arms, leaving four arms without platforms in addition to the start arm. Thus, like the 8-Arm maze, the 12-Arm maze had 7 arms platformed. This was done to ensure an equal number of working memory items across both mazes, such that the working memory information that needed to be handled increased in equal increments across trials for both the 8-Arm maze and the 12-Arm maze. In the 8-Arm task, there was a start arm and no reference memory arms, whereas in the 12-Arm task there was a start arm plus four reference memory arms. This allowed for a rigorous and systematic evaluation of the addition of reference memory to a working memory task. The temperature of the water was likewise kept at a range of 18–20°C. Each subject had unique platform locations that were semi-randomly determined and remained fixed for each animal across days throughout the experiment. Salient external cues were placed in the testing room, including two panels on the walls outside the maze, the door, a row of tables with heating lamps, and the experimenter at the start arm.

The testing procedure for the 12-Arm task was identical to the testing procedure for the 8-Arm task. WMC errors were the number of entries into an arm that previously contained a platform for that session. Reference Memory (RM) errors were the number of first entries into an arm that has never contained a platform for that particular animal, referred to as a reference memory arm (this included the start arm). Working Memory Incorrect (WMI) errors were any subsequent entry into an arm that had never contained a platform; in this manner, WMI errors are a working memory failure by repeatedly entering a reference memory arm in the search for an escape platform. Total errors were the sum of all errors made across a trial. Errors were evaluated for each session and were analyzed across all days of testing.

### Visible Platform Task

After WRAM testing was completed, rats received 1 day of testing on the VP task as previously described (Braden et al., 2010; Bimonte-Nelson, 2015b; Koebele et al., 2017; Prakapenka et al., 2018), to ensure that all subjects were able to successfully complete the procedural components of a water-escape task. Rats were placed into a rectangular tub (100 × 60 cm) with water kept at 18–20°C, and external maze cues were covered by an opaque curtain that encircled the maze. Subjects were given 90 s to locate a clearly visible black platform protruding 4 cm from the water throughout testing. Once the platform was found, the rats remained there for 15 s before being returned to its warm home cage with an inter-trial interval of 5–8 min. Subjects were given six trials, with the location of the platform varying in space semi-randomly, and the drop-off location remaining constant across trials. Performance on the VP task was evaluated *via* latency (seconds) to the platform.

### Tissue Collection

The day following the conclusion of behavioral testing, rats were deeply anesthetized using isoflurane, decapitated, and brains were rapidly dissected and frozen. The dissected tissues were weighed in pre-weighed microcentrifuge tubes and stored at −70°C until analysis commenced. Dissections were performed using ice cold saline for the following brain regions as described by Paxinos and Watson (1998): frontal cortex (plates 5–14), perirhinal cortex (plates 39–42), entorhinal cortex (EC; plates 39–42), the dorsal hippocampus (plates 33–35), and the ventral hippocampus (plates 39–42). For each brain, the frontal cortex was first collected from the dorsal portion of the whole brain. Next, the brain was cut in the coronal plane and the dorsal hippocampus was collected. Finally, a secondary coronal cut was made posterior to the first cut, where the ventral hippocampus and the entorhinal and perirhinal cortices were collected.

### ChAT Activity Analyses

To prepare brain tissues collected at sacrifice for ChAT activity analyses, cold sonication medium containing 10 mM EDTA and 0.5% Triton X-100 was prepared and added in a ratio of 10 μl solution per 1 mg tissue, where tissue weight was measured as a wet weight during sacrifices; each sample was then sonicated. After sonication, each sample was aliquoted and assayed to ascertain its total protein concentration (Bradford, 1976). The ChAT activity of each sample was determined using a radioenzymatic method that has previously been published (Gibbs et al., 2011), wherein the total production of [^3^H]-acetylcholine was measured under a 37°C reaction for 30 min in a medium with a final acetyl-CoA concentration of 0.25 mM (30,000–40,000 d.p.m./tube; Sigma Inc., St. Louis, MO), with 10 mM EDTA, 10 mM choline chloride, 0.2 mM physostigmine-sulfate, 300 mM NaCl, and 50 mM sodium phosphate buffer (7.4 pH). The determination of background was performed by processing identical tubes without the addition of sample. Reactions were terminated in 4 mL 10 mM cold sodium phosphate buffer, then with the addition of 1.6 mL acetonitrile containing 5 mg/mL tetrephenylboron. Phase separation was obtained by adding 8 mL of Econo-Flour scintillation cocktail (Packard Instruments, Meriden, CT) and by counting the total cpm while in the organic phase. The difference between the total cpm and the background cpm was then utilized to estimate the total amount of ACh produced per sample. Aliquots of each diluted sample was used in determining the total protein in each reaction tube, as well as for collecting the total ChAT activity. The average ChAT activity was calculated from three reaction tubes per sample, where ChAT activity was represented as pmol of ACh synthesized/hr/μg protein.

### Statistical Analyses

For behavior assessments, data were analyzed using an omnibus repeated measures ANOVA with Maze (two levels: 8-Arm and 12-Arm) and Age (two levels: Young and Aged) as independent variables, Errors as the dependent variable, and Trials within Days as repeated measures. While 12 days of baseline testing were conducted, Day 1 of WRAM testing was excluded from all analyses. On Day 1, animals are water-maze naïve; therefore, the first day of testing is considered to be a training session and is typically excluded from subsequent analyses (Bimonte-Nelson et al., 2015; Hiroi et al., 2016). As an initial assessment, to evaluate overall performance on the WRAM, omnibus repeated measures ANOVAs were run to examine the main effects of Age and Maze on Total errors across Days 2–12 of testing. Similar analyses were run for WMC errors for both mazes. In addition, for the 12-Arm WRAM only, as it was the only task with a reference memory component, we analyzed RM errors (first entry into a reference memory arm) and WMI errors (repeat entries into these reference memory arms). Based on prior publications from our laboratory concerning blocking (Bimonte-Nelson et al., 2015), particularly those involving the 12-Arm WRAM (Bimonte et al., 2003), days of WRAM testing were blocked into two separate phases to distinguish the learning and memory retention portions of testing: Days 2–7 were the Acquisition Phase of testing, and Days 8–12 were the Asymptotic Phase of testing. Each phase of testing was analyzed separately using repeated measures ANOVA for WMC errors for both the 8-Arm and 12-Arm mazes, as well as for WMI and RM errors for the 12-Arm WRAM. RM errors by definition are capped at 5 total errors within a testing day (the first entry into each of the 5 RM arms), making performance for a given trial heavily dependent on performance during previous trials for this measure. Therefore, main effects of Age were analyzed for this measure, but effects of Trial or interactions with Trial were not probed, as is the common procedure in our laboratory (Bimonte-Nelson et al., 2004; Braden et al., 2015, 2017; Mennenga et al., 2015c). In the case of a significant Age × Maze interaction for WMC errors, the variables Age and Maze were analyzed separately as repeated measures ANOVA, with Maze or Age as the independent variable, Errors as the dependent variable, and Trials within Days as the repeated measure. In the case of a significant Trial × Maze, Trial × Age, or Trial × Age × Maze interaction for either testing phase, all trials were analyzed individually as repeated measures ANOVAs, with Maze and/or Age as the independent variables and Days as the repeated measure. Upon significant Age × Maze interactions for an individual trial, the variables Age and Maze were analyzed separately as repeated measure ANOVAs, with Maze or Age as the independent variable, Errors as the dependent variable, and Days as the repeated measure. For all behavioral analyses, alpha was set at *p* < 0.05, and marginal findings were set at *p* values between 0.05 and 0.10.

Pearson *r* correlations were performed, along with Fisher's tests of significance, to determine the relationship between RM errors made in earlier trials (T2) with WMC errors made on the highest working memory load trial (T7) on Day 12 for the 12-Arm WRAM, with young and aged groups undergoing separate analysis. This was performed to determine if the reference memory errors made earlier within a testing day correlated with a greater number of WMC errors made at the end of the testing day; such a relationship would indicate that reference memory ability early within a session impacts working memory ability at the end of a session, when working memory load is highest.

ChAT activity was analyzed for the dorsal and ventral hippocampus, frontal cortex, entorhinal cortex, and perirhinal cortex using a series of ANOVAs with Age and Maze as the independent variables and ChAT activity as the dependent variable (pmol of ACh produced/hr/μg protein).

Pearson *r* correlations were performed with Fisher's tests of significance to determine the relationship between ChAT activity and WMC errors for the Asymptotic Phase (Days 8–12), which were averaged across days and summed across trials. These correlations were run for each of the four groups separately (8-Arm Young, 8-Arm Aged, 12-Arm Young, 12-Arm Aged).

## Results

### Water Radial-Arm Maze

#### Overall WRAM Performance

Maze components included seven spatial working memory arms in both the 8-Arm and 12-Arm mazes, plus the addition of reference memory arms in only the 12-Arm maze. WMC errors (entries into previously platformed arms, referred to as working memory arms) were analyzed for both mazes, and the unique addition of reference memory arms in the 12-Arm WRAM yielded analysis of RM errors (first entries into unplatformed arms, referred to as reference memory arms) and WMI errors (re-entries into unplatformed, or reference memory, arms). To aid the collective interpretation, we visually represent the proportion of WMC errors that contribute to Total errors from the 8-Arm WRAM and the 12-Arm WRAM; we did this for each age group separately (Figure 2). As expected, the majority of Total errors in the 8-Arm WRAM were WMC errors; however, on the 12-Arm WRAM, WMC errors accounted for only a portion of Total errors, likely due to the presence of the reference memory arms which give rise to the potential to commit additional error types.

Analysis of maze performance revealed a main effect of Day for Total errors [*F*_(10, 350)_ = 2.28, *p* < 0.05], with errors decreasing across Days 2–12, indicating that rats did indeed learn the WRAM task (data not shown). For Total errors [*F*_(1, 35)_ = 318.11, *p* < 0.0001], as well as WMC errors [*F*_(1, 35)_ = 46.91, *p* < 0.0001], there was an effect of Maze across Days 2–12, whereby rats in the 12-Arm WRAM made more errors than those in the 8-Arm WRAM. This finding indicates that the addition of a reference memory component in the 12-Arm WRAM resulted in rats making more WMC errors as compared to rats tested on the 8-Arm WRAM, which does not contain this additional reference memory component (data not shown). There also was a main effect of Age for Total errors across Days 2–12 [*F*_(1, 35)_ = 17.35, *p* < 0.001; data not shown], indicating that aged rats made more errors than young rats. This main effect of Age was also seen in WMC errors [*F*_(1, 35)_ = 8.41, *p* < 0.01; data not shown], for Days 2–12, such that aged rats made more WMC errors than did young rats, regardless of maze. Main effects of Age for RM errors [*F*_(1, 18)_ = 5.22, *p* < 0.05] and WMI errors [*F*_(1, 18)_ = 14.69, *p* < 0.01] for rats tested on the 12-Arm WRAM exclusively were also detected across Days 2–12 (data not shown).

#### Working Memory Performance for the Acquisition Phase (Days 2–7)

**Figure 3:**
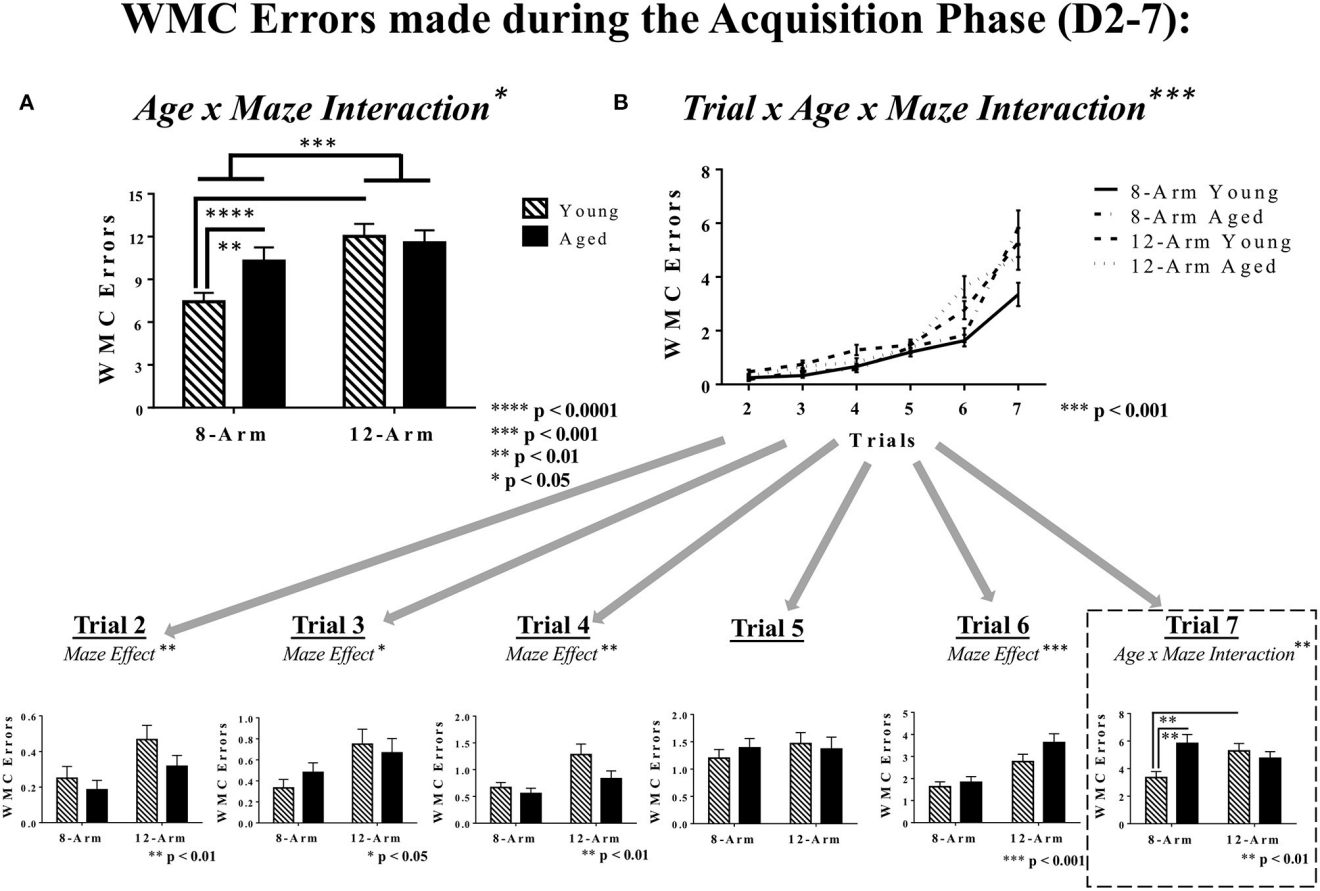
WMC errors during the Acquisition Phase of WRAM testing. **(A)** WMC errors for the Acquisition Phase of WRAM testing (Days 2–7). With an effect of Maze and an Age × Maze Interaction, it was found that within the 8-Arm WRAM there was an effect of Age, but not for the 12-Arm WRAM; additionally, young rats tested on the 8-Arm WRAM performed better than those tested on the 12-Arm WRAM, but performance across maze did not differ for aged rats. **(B)** Working memory performance as measured by WMC errors made across all testing trials (Trials 2–7). At the highest working memory load (Trial 7), there was an Age × Maze interaction, where aged rats performing on the 8-Arm WRAM made more errors than their younger counterparts, and the rats performing on the 12-Arm WRAM did not differ by age. Additionally, for young rats there was an effect of Maze, where rats tested on the 8-Arm WRAM made fewer errors than their counterparts tested on the 12-Arm WRAM, with no effect amongst aged rats.

Analysis of performance during the Acquisition Phase revealed a main effect of Maze [*F*_(1, 35)_ = 13.70, *p* < 0.001], whereby rats tested in the 12-Arm WRAM performed worse than those in the 8-Arm WRAM (Figure 3A), as well as a significant Age × Maze interaction [*F*_(1, 35)_ = 4.31, *p* < 0.05]. Further analyses showed that within the 8-Arm WRAM, aged rats performed worse than young rats [*F*_(1, 17)_ = 9.68, *p* < 0.01], but within the 12-Arm WRAM there was no difference in performance with age. For young rats, there was a main effect of Maze for the Acquisition Phase [*F*_(1, 18)_ = 30.84, *p* < 0.0001], with young rats tested on the 8-Arm WRAM performing better than those tested on the 12-Arm WRAM. No significant effect of Maze was seen for aged rats. Significant interaction effects for Trial × Age [*F*_(5, 175)_ = 2.88, *p* < 0.05], Trial × Maze [*F*_(5, 175)_ = 3.25, *p* < 0.01], and Trial × Age × Maze [*F*_(5, 175)_ = 5.28, *p* < 0.001] were also found during the Acquisition Phase, indicating that rats performed differently throughout the series of trials depending on age and maze (Figure 3B). Subsequent analyses of each trial alone showed that the rats tested on the 12-Arm WRAM performed worse than those tested on the 8-Arm WRAM across the majority of trials, suggesting that the presence of a reference memory component impacted performance regardless of whether the working memory load demand was low or high. Specifically, main effects of Maze were found on Trial 2 [*F*_(1, 35)_ = 8.10, *p* < 0.01], Trial 3 [*F*_(1, 35)_ = 5.41, *p* < 0.05], Trial 4 [*F*_(1, 35)_ = 8.23, *p* < 0.01], and Trial 6 [*F*_(1, 35)_ = 17.82, *p* < 0.001]. For the highest working memory load trial, Trial 7, there was an Age × Maze interaction [*F*_(1, 35)_ = 8.21, *p* < 0.01], with aged rats tested on the 8-Arm WRAM making more errors on Trial 7 than young rats on the 8-Arm WRAM [*F*_(1, 17)_ = 13.74, *p* < 0.01]. In contrast, no age effects on Trial 7 were present for the 12-Arm WRAM. Analysis of the performance of young rats on Trial 7 during the Acquisition Phase revealed an effect of Maze, whereby young rats tested on the 12-Arm WRAM made more errors than those tested on the 8-Arm WRAM [*F*_(1, 18)_ = 9.92, *p* < 0.01]; this effect was not found for aged rats (Figure 3B).

#### Working Memory Performance for the Asymptotic Phase (Days 8–12)

**Figure 4:**
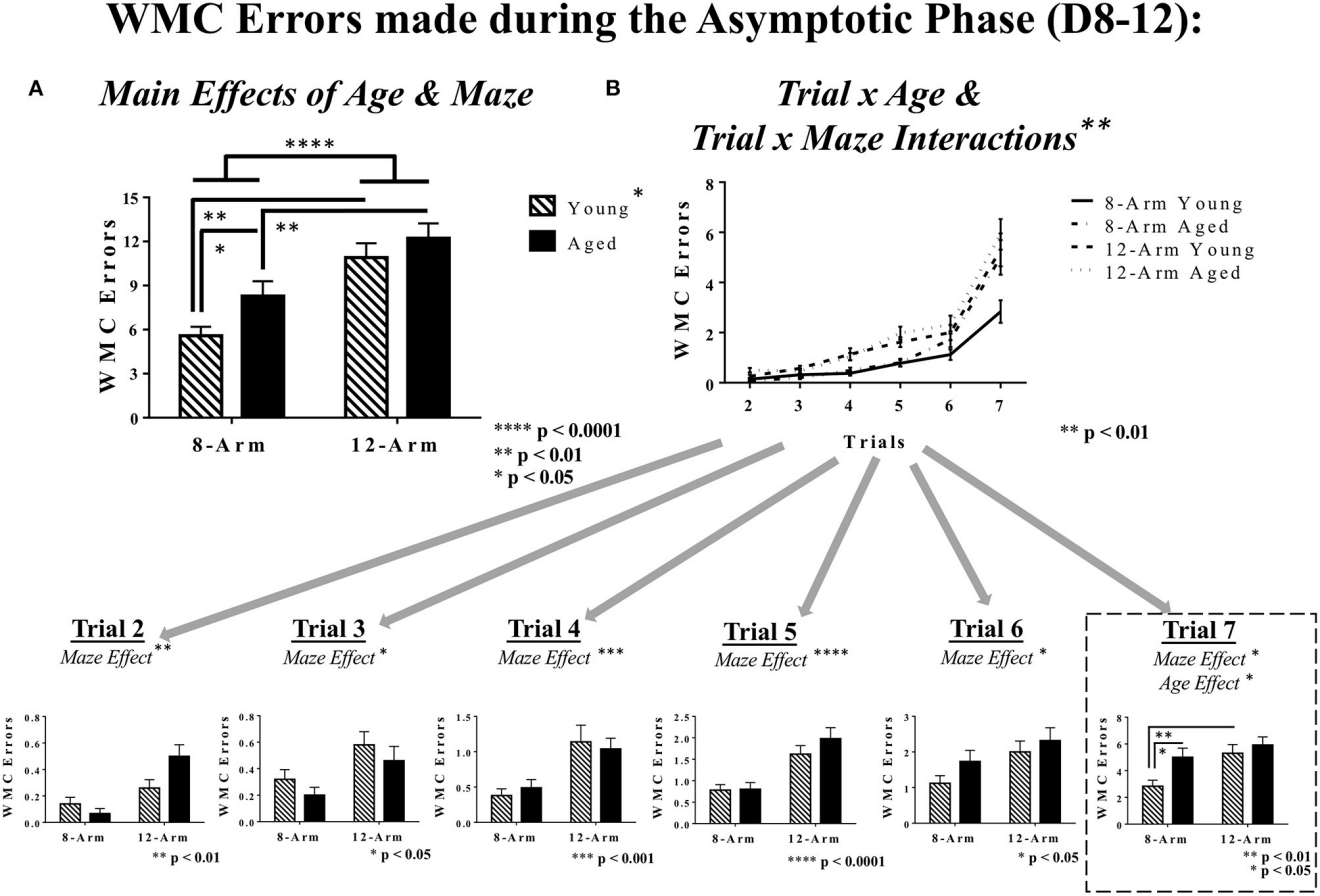
WMC errors during the Asymptotic Phase of WRAM testing. **(A)** WMC errors for the Asymptotic Phase of WRAM testing (Days 8–12). With an effect of Maze and an effect of Age, it was found that within the 8-Arm WRAM there was an effect of Age, but not for the 12-Arm WRAM; notably distinct from the Acquisition Phase, both young and aged rats tested on the 8-Arm WRAM performed better their counterparts tested on the 12-Arm WRAM. **(B)** Working memory performance as assessed *via* WMC errors made across all testing trials (Trials 2–7). At the highest working memory load (Trial 7), there was a main effect of Maze and a main effect of Age, where aged rats performing on the 8-Arm WRAM made more errors than their younger counterparts, and the rats performing on the 12-Arm WRAM did not differ by age. Additionally, there was an effect of Maze for young rats exclusively, where young rat performance on the 8-Arm WRAM was better than that of the 12-Arm WRAM, with no effect of Maze for aged rats.

Analysis of performance during the Asymptotic Phase revealed a main effect of Age [*F*_(1, 35)_ = 4.40, *p* < 0.05], whereby aged rats made more working memory errors than young rats, and a main effect of Maze [*F*_(1, 35)_ = 23.21, *p* < 0.0001], where rats tested on the 12-Arm WRAM, evaluating both working and reference memory, made more errors than those tested on the 8-Arm WRAM, evaluating solely working memory (Figure 4A). While there was not a significant Age × Maze interaction for the Asymptotic Phase, due to the nature of the relationships between Age and Maze that we noted during the Acquisition Phase, these same analyses were conducted within the Asymptotic Phase. Within the 8-Arm WRAM there was a main effect of Age [*F*_(1, 17)_ = 4.68, *p* < 0.05], whereby aged rats made more errors than their young counterparts. This was not true of rats tested on the 12-Arm WRAM. Furthermore, there were effects of Maze for both young [*F*_(1, 18)_ = 13.64, *p* < 0.01] and aged [*F*_(1, 17)_ = 9.79, *p* < 0.01] rats, with rats of each age tested on the 12-Arm WRAM making more WMC errors than those tested on the 8-Arm WRAM. There also were interactions for both Trial × Age [*F*_(5, 175)_ = 3.49, *p* < 0.01] and Trial × Maze [*F*_(5, 175)_ = 3.25, *p* < 0.01], indicating that, similar to the Acquisition Phase of testing, performance during the Asymptotic Phase differed across trials depending upon age and maze (Figure 4B). Subsequent analyses of each testing trial revealed a main effect of Maze [Trial 2: *F*_(1, 35)_ = 11.10, *p* < 0.01; Trial 3: *F*_(1, 35)_ = 6.90, *p* < 0.05; Trial 4: *F*_(1, 35)_ = 14.36, *p* < 0.001; Trial 5: *F*_(1, 35)_ = 20.94, *p* < 0.0001; Trial 6: *F*_(1, 35)_ = 7.35, *p* < 0.05; Trial 7: *F*_(1, 35)_ = 6.75, *p* < 0.05], with rats tested on the 12-Arm WRAM making more errors than those tested on the 8-Arm WRAM. For the highest working memory load trial (Trial 7), there was a main effect of Age [*F*_(1, 35)_ = 4.56, *p* < 0.05] and a significant effect of Maze [*F*_(1, 35)_ = 6.75, *p* < 0.05, as mentioned previously], where aged rats made more errors than young rats, and rats tested in the 12-Arm WRAM made more errors than those tested on the 8-Arm WRAM. Much like in the Acquisition Phase of testing, there was an effect of Maze for young rats, where young rats tested on the 8-Arm WRAM performed better than those tested on the 12-Arm WRAM [*F*_(1, 18)_ = 8.99, *p* < 0.01], and this Maze effect was not present for aged rats. Regarding an effect of Age within the solely working memory task, aged rats tested on the 8-Arm WRAM performed worse than young rats tested on the 8-Arm WRAM [*F*_(1, 17)_ = 4.92, *p* < 0.05]. The two ages did not differ for WMC errors on the 12-Arm WRAM. The presence of an age effect for WMC errors in the 8-Arm WRAM, but not the 12-Arm WRAM, indicates that the addition of a reference memory component in the 12-Arm WRAM overpowered any age-related differences for this evaluation of working memory (Figure 4B).

Collectively, these data suggest that, regardless of phase of learning, the addition of a reference memory component in the 12-Arm WRAM resulted in both young and aged rats making a larger number of WMC errors compared to the 8-Arm WRAM, where multiple reference memory arms were not present. Thus, for the 12-Arm WRAM, age did not appear to play a significant role for WMC performance, as both young and aged rats displayed poor working memory performance for this measure. However, for the 8-Arm WRAM, age played a significant role, whereby age-related deficits were seen with an increasing working memory load for WMC errors.

#### Assessment of Age-Related Differences in Working Memory and Reference Memory Performance When Reference Memory Requirements Are Added

After addressing the differences in performance between the 8-Arm WRAM and the 12-Arm WRAM for both young and aged rats using WMC errors, the only measure shared by both mazes, the reference memory arm component of the 12-Arm WRAM was analyzed alone to determine age-related outcomes. Thus, within the 12-Arm WRAM, performance was additionally quantified *via* assessment of first entries into arms that never contained a platform (RM errors) and repeat entries into arms that never contained a platform (WMI errors). In this manner, we were able to evaluate the unique effects that the addition of reference memory arms had on both reference memory and working memory performance for both aged and young rats.

When evaluating RM errors in the Acquisition Phase, the main effect of Age was not significant, indicating that young and aged rats did not differ in maintaining reference memory items while learning the task. For the Asymptotic Phase, there was a main effect of Age [*F*_(1, 18)_ = 5.47, *p* < 0.05] (data not shown), whereby aged rats made more RM errors than young rats, suggesting that young rats better maintained reference memory information at the end of testing on the maze that evaluated both working and reference memory.

**Figure 5:**
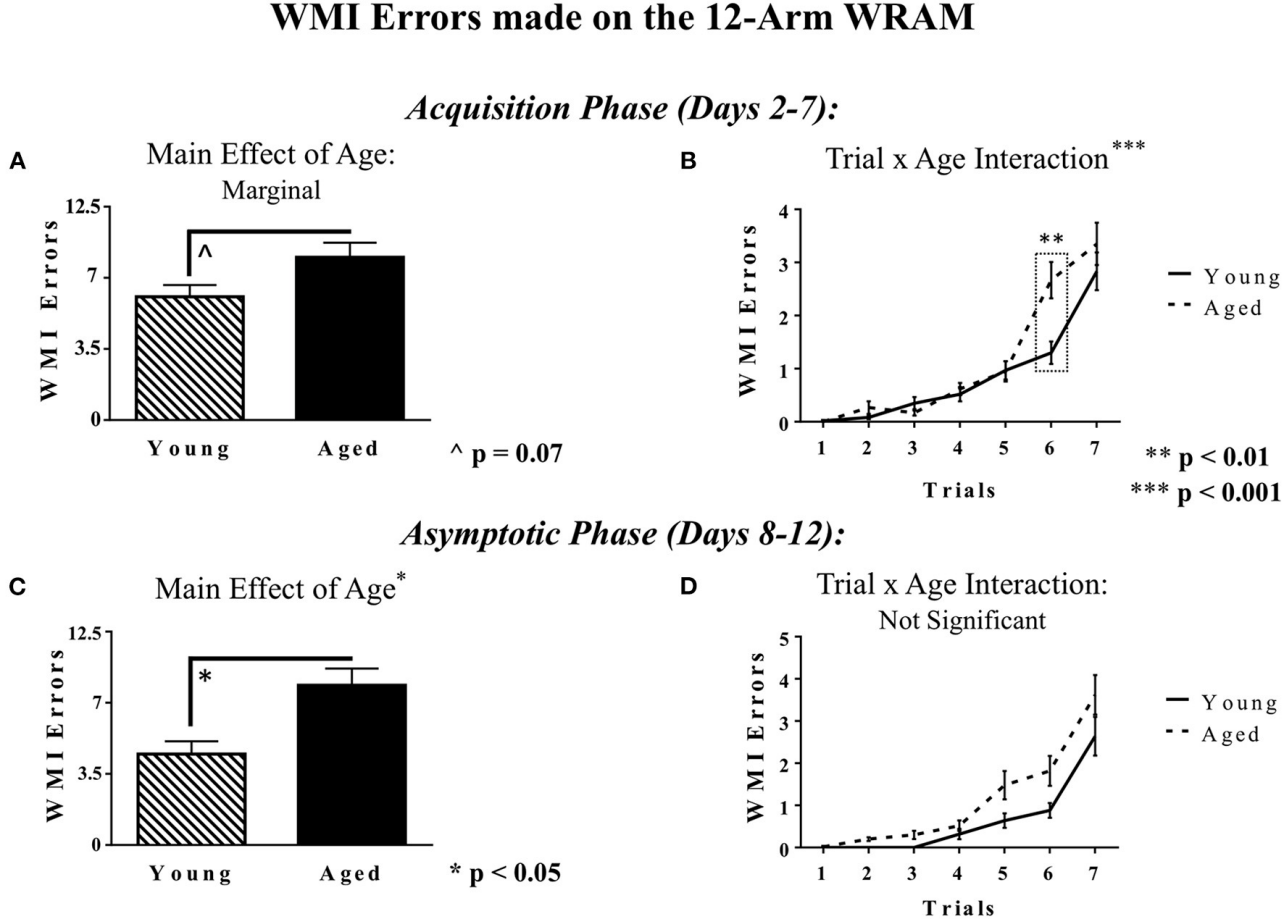
WMI errors within the 12-Arm WRAM. **(A)** The marginal main effect of Age on WMI errors within the 12-Arm WRAM for the Acquisition Phase (Days 2–7), where aged rats made marginally more WMI errors than young rats. **(B)** WMI errors across Trials 1–7 for Acquisition Phase (Days 2–7) of testing. **(C)** The main effect of Age on WMI errors within the 12-Arm WRAM for the Asymptotic Phase of testing (Days 8–12), where aged rats made significantly more WMI errors than young rats. **(D)** WMI errors across Trials 1–7 for the Asymptotic Phase (Days 8–12) of testing. For the Acquisition Phase, age effects were particular to a high working memory load trial, whereas by the end of testing, in the Asymptotic Phase, the age effects were not trial-dependent.

For WMI errors, during the Acquisition Phase, there was a marginal main effect of Age [*F*_(1, 18)_ = 3.81, *p* = 0.07], whereby aged rats tended to make more WMI errors while learning the task than did young rats (Figure 5A). Additionally, during the Acquisition Phase, there was a significant Trial × Age interaction [*F*_(6, 108)_ = 4.46, *p* < 0.001; Figure 5B], indicating that the effects of Age on WMI errors were trial-dependent. Further analyses demonstrated that on Trial 6, with a high working memory load, there was an effect of Age, whereby aged rats made more entries into unplatformed arms than young rats [*F*_(1, 18)_ = 14.70, *p* < 0.01]. Likewise, across the Asymptotic Phase (Days 8–12), there was a main effect of Age [*F*_(1, 18)_ = 6.30, *p* < 0.05], where aged rats made more WMI errors than young rats, displaying that the working memory capabilities of aged rats suffered relative to young rats with the addition of a reference memory component (Figure 5C). The Trial × Age interaction was not significant (Figure 5D).

#### Correlations Between Reference Memory and Working Memory in the 12-Arm WRAM

**Figure 6:**
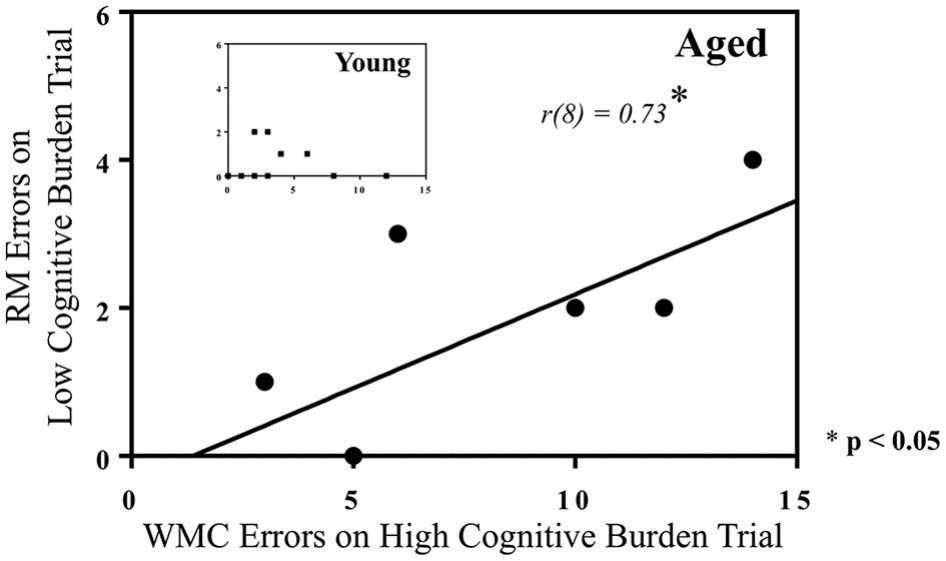
Correlations between RM and WMC errors on the WRAM. A significant positive correlation was found between RM errors made on an early trial (Trial 2), with a relatively low cognitive load, and WMC errors made on the last trial (Trial 7), or the highest cognitive load trial, for aged rats performing on the 12-Arm WRAM on the last day of testing, but not for young rats [*R*^2^ = 0.53, *r*(8) = 0.73, *p < 0.05]. Several rats in the aged group made the same number of RM and WMC errors for this figure, resulting in the overlap of data points represented.

To further investigate the role that the addition of a reference memory component to a working memory task plays in working memory performance in young and aged rats, a correlation was performed for each age separately between RM errors on Day 12 for Trial 2, and WMC errors on Day 12 for Trial 7, in the 12-Arm WRAM. In this manner, we evaluated whether reference memory errors made on the earliest trial were related to working memory errors made on the last trial, when working memory load was most taxed. This allowed for evaluation of the role that reference memory prowess, demonstrated early within a testing day, played in later working memory outcomes. There was a positive correlation for the aged group [*r*(8) = 0.73, *p* < 0.05; Figure 6], indicating that aged rats that made more reference memory errors on an early trial tended to make more working memory errors when working memory was most burdened. There was no correlation for young rats. This further indicates the non-orthogonality of reference memory and working memory in the WRAM task, particularly for aged rats when working memory is sufficiently taxed.

### Visible Platform Task

**Figure 7:**
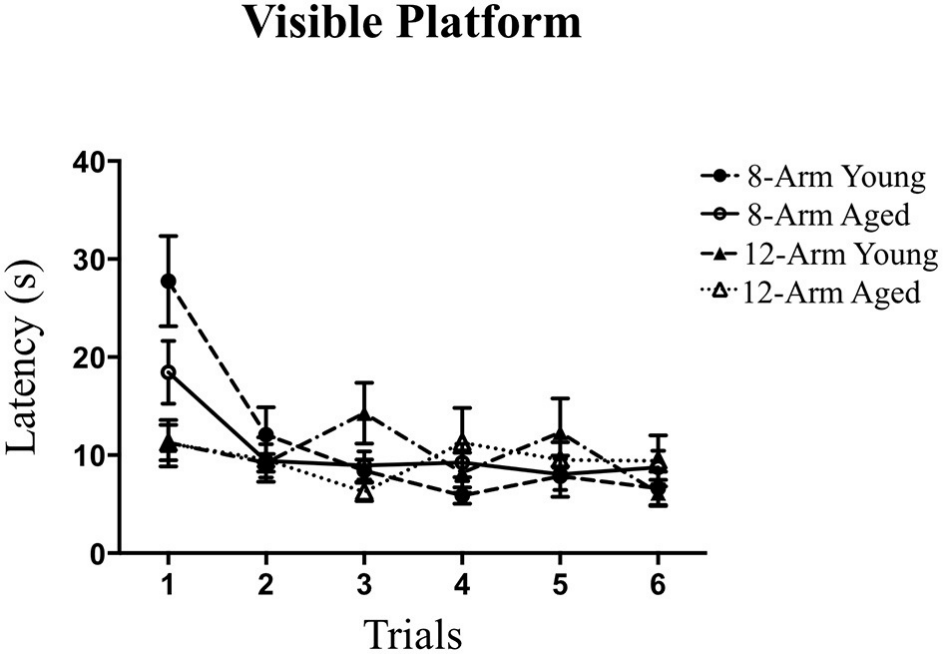
Performance on the Visible Platform (VP) task. Latency (in seconds) across Trials 1–6 on the VP task, where groups did not differ in ability to perform the procedural components of a water escape task.

Motor and visual acuity for solving the procedural components of a water-escape task were analyzed using the VP task. Groups did not differ from each other across testing trials in the ability to locate and navigate to the visible platform following the first introductory trial [Trials 2–6; Effect of Age: *F*_(1, 35)_ = 0.01, *p* = n.s.; Effect of Maze: *F*_(1, 35)_ = 0.89, *p* = n.s.; Age × Maze Interaction: *F*_(1, 35)_ = 0.43, *p* = n.s.]. The average escape latency for each group by the final trial was no greater than 10 s, indicating that rats had the visual and motor acuity necessary to complete a water escape task (Figure 7). Given that our laboratory has shown that this task identifies rats with motor issues (Mennenga et al., 2015a), the findings here indicate that the young and aged rats in this experiment did not have significant motor issues that would hinder maze performance.

### ChAT Activity Analyses

**Figure 8:**
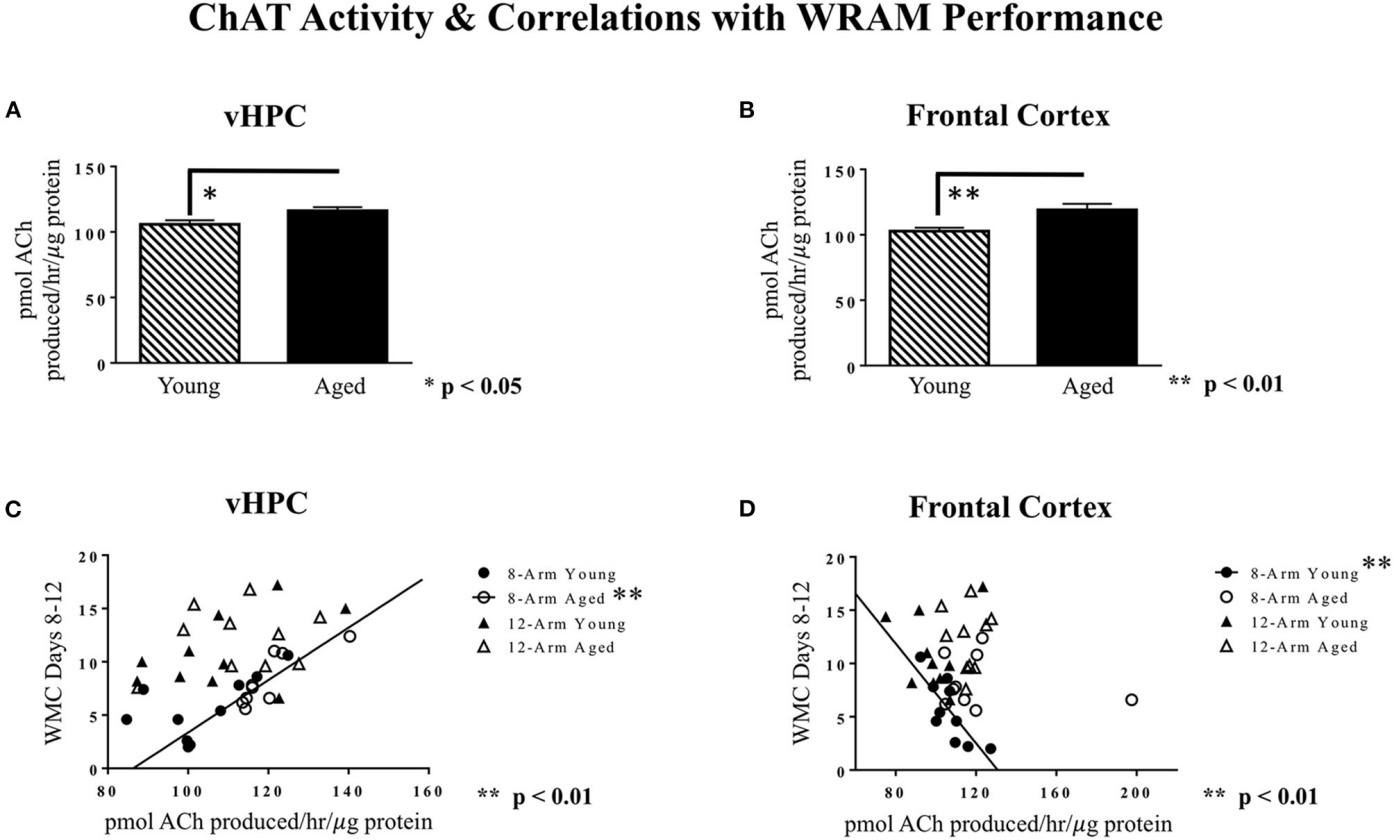
ChAT activity in various brain regions, and correlations with WRAM performance. **(A)** Average ChAT activity levels in the ventral hippocampus, where aged rats had greater ChAT activity. **(B)** Average ChAT activity levels in the frontal cortex, where aged rats had greater ChAT activity. **(C)** A significant positive correlation was found between ChAT activity in the ventral hippocampus and WMC errors made during the Asymptotic Phase for aged rats performing on the 8-Arm WRAM specifically [*R*^2^ = 0.70, *r*(7) = 0.84, **p < 0.01). **(D)** A significant negative correlation was found between ChAT activity in the frontal cortex and WMC errors made during the Asymptotic Phase for young rats tested on the 8-Arm WRAM [*R*^2^ = 0.61, *r*(8) = −0.78, **p < 0.01].

ChAT activity was analyzed for the dorsal hippocampus, ventral hippocampus, frontal cortex, perirhinal cortex, and entorhinal cortex. For the ventral hippocampus and the frontal cortex, aged rats exhibited higher levels of ChAT activity than young rats [main effect of Age for ventral hippocampus: *F*_(1, 35)_ = 6.30, *p* < 0.05; main effect of Age for frontal cortex: *F*_(1, 35)_ = 9.56, *p* < 0.01; Figures 8A,B]. For the dorsal hippocampus, entorhinal cortex, and perirhinal cortex, there were no main effects of Age (data not shown). There were no main effects of Maze, nor were there any Age × Maze interactions, for any of the brain regions analyzed.

Correlations between ChAT activity levels in each brain region and WRAM performance (WMC errors made averaged across Days 8–12) were analyzed to further investigate the relationships between these variables, and to assess whether these subsequent relationships vary by age and memory type. For aged rats tested on the 8-Arm WRAM, there was a positive correlation between ChAT activity in the ventral hippocampus and WMC errors [*r*(7) = 0.84, *p* < 0.01; Figure 8C], whereby more working memory errors made specifically on the 8-Arm WRAM correlated with higher levels of ventral hippocampus ChAT activity for aged rats. For young rats tested on the 8-Arm WRAM, the opposite relationship was found for the frontal cortex; indeed, there was a negative correlation between frontal cortex ChAT activity and WMC errors [*r*(8) = −0.78, *p* < 0.01; Figure 8D], whereby higher ChAT activity in the frontal cortex in young rats was correlated with fewer working memory errors on the 8-Arm WRAM.

## Discussion

The current study demonstrated that the addition of a reference memory component impaired working memory performance in both young and aged female rats throughout testing on the WRAM task. However, the type of memory failure that increased after the addition of the reference memory component differed between the ages. Specifically, aged rats made more working memory errors than young rats on the working memory only task, and these comparably higher working memory errors in aged rats persisted when reference memory was added to the task. Aged rats evaluated on the combined working and reference memory task also made more reference memory errors than young rats. These effects were pronounced for the latter portion of testing. Thus, young rats learned to successfully handle the additional reference memory challenge, while aged rats did not. This yielded an age-related failure to maintain reference memory information in the combined working and reference memory task.

Even though the number of working memory arms was constant in both the sole working memory task and the combined working and reference memory task, there were age-related impairments in the ability to handle an increased working memory load only in the task that tested exclusively working memory. Thus, the addition of a reference memory challenge further burdened the ability to handle an increasing working memory load in young rats, while in aged rats, the working memory load was likely already taxed maximally even without the reference memory challenge. These findings correspond to previous work demonstrating age-related deficits in performance on a multitude of spatial learning and memory tasks (Frick et al., 1995; Bimonte et al., 2003; Coppola et al., 2014, 2015; Koebele et al., 2017). Our current findings also show that the presence of both a reference memory and working memory component combined with a sufficient working memory load results in a working memory impairment in young rats that is comparable to the impairment in aged rats when they are tested solely on a working memory task. Overall, both young and aged rats were able to learn the procedural components of the WRAM, as shown by their improved performance across all days of WRAM testing. Additionally, young and aged rats did not differ in their ability to perform the procedural components of a water-escape task as tested in the VP task, suggesting that differences in WRAM scores were not attributable to differences in visual or motor competencies necessary to complete this battery.

For Total errors, there was an effect of Age across all testing days, whereby aged rats made more errors of each memory type than did young rats. This indicated that, regardless of maze type, aged rats were less able to handle the cognitive complexity of the spatial memory task than their younger counterparts; this age-effect is a replication and extension of prior work showing age-related impairments in spatial learning and memory (Barnes, 1979; Barnes et al., 1980; Frick et al., 1995; Bimonte et al., 2003; Shukitt-Hale et al., 2004). These findings also complement the human literature, wherein aged individuals demonstrate poorer spatial navigation as compared to young individuals, which largely stems from a deficit not in encoding or recognizing landmarks, but in the temporospatial ordering of these landmarks (Wilkniss et al., 1997). Even though both mazes had seven working memory arms to remember, overall maze performance was poorer when reference memory arms were added on to the demand. Specifically, rats tested on the combined working and reference memory 12-Arm WRAM made more Total errors into those 7 working memory arms than rats tested on the 8-Arm WRAM. Notably this effect occurred regardless of age such that in both young and aged rats, spatial learning and memory was more heavily taxed by the addition of a reference memory component on the WRAM task.

Analyses of performance at the highest working memory load demonstrated unique maze effects between young and aged rats. When comparing WMC errors in both testing phases at the highest working memory load trial, young rats made fewer errors on the working memory only task as compared to the task that evaluated reference memory as well, as noted by the Maze effect in young rats. In contrast, there was no Maze effect for aged rats, indicating that the addition of a reference memory component did not further increase working memory impairments in the aged cohort. These findings support the tenet that the sole spatial working memory task is so challenging for aged rats that the addition of a reference memory component does not further burden working memory. This phenomenon likely contributed to the emergence of age-related effects that differed depending on maze type. Indeed, for both testing phases, at the highest working memory load, aged rats tested in the working memory only task made more errors on the pure working memory measure than young rats. In contrast, at the highest working memory load, young and aged rats did not differ in their ability to maintain working memory information on the combined working and reference memory task, suggesting that adding a reference memory component impacted the observation of age-related effects.

On the working memory only task there were age effects on the pure working memory measure, while on the combined working and reference memory task, the profile of age-related decrements shifted from pure working memory to first and repeat failures of reference memory information, the latter of which can be defined as a working memory process. While there were no age effects on entries into working memory arms (WMC errors) when both working and reference memory were present, there was an age-related deficit on both first and repeat entries into reference memory arms (RM and WMI errors). These results demonstrate that aged rats were not only challenged by the presence of 7 working memory arms, which were found in both mazes, but that the addition of reference memory arms led to additional working memory failures in revisiting these reference memory arms, a challenge that young rats were able to learn across testing. These findings suggest that there are differences in the manner with which young and aged female rats experience or solve this complex reference and working memory task. In rodents and other species, aging can impact strategies used to solve spatial memory tasks (Barnes et al., 1980; Begega et al., 2001; Rodgers et al., 2012; Coppola et al., 2014, 2015); it is possible that the differential outcomes for young and aged rats in the current study are the result of different strategies utilized to solve this complex water-escape task. Such findings have also been observed following manipulations in ovarian hormone levels in female rodents (Korol and Kolo, 2002); this is critical, as gonadal hormone levels change across the lifespan in the female rat, and could result in differential strategy utilization as a function of age (Korol and Pisani, 2015).

Measuring both working and reference memory simultaneously in the 12-Arm WRAM provided the unique opportunity to determine if rats that excelled in reference memory when there was minimal working memory demand (Trial 2) also excelled in working memory when demand was maximal (Trial 7). Thus, we asked, does higher reference memory ability relate to higher working memory ability at its highest load? Our results showed that this relationship was present in aged rats, but not in young rats. Specifically, for aged rats, there was a significant positive correlation, with aged animals that made more reference memory errors tending to also make more working memory errors at the highest challenge. This positive relationship between the two error types further supports the tenet that the two measures are not orthogonal in aged rats, presenting an exciting opportunity for further investigation into the relationship between working and reference memory, and putative age-related differences in this relationship. For young rats, these two error types did not correlate. One explanation for this is that young animals made very few reference memory errors on the last day of baseline testing (Day 12), yielding little variability within the group. It therefore seems likely that while the measures of reference memory and working memory are logistically scored and quantified independently for the WRAM, they are not functionally orthogonal from a psychophysiological systems approach. Probing and deciphering reciprocal relationships between these memory types will enrich and diversify analytic interpretations of maze data, especially in age-related contexts. That the presence of working memory might impact reference memory outcomes should be carefully considered. For example, systematically examining reference memory when tested in concert with working memory, as done here with the WRAM, as compared to testing on a task where the sole measure is reference memory, as done with the win-stay Morris Maze, can aid interpretation of not only reference memory findings, but relationships amongst working and reference memory as working memory demand shifts from minimal to maximal difficulty. Indeed, reference memory outcomes on the WRAM (when working memory is also present) often do not parallel reference memory outcomes on the Morris Maze (when working memory is not present), for example, with pharmaceutical or genetic manipulations (Acosta et al., 2010; Camp et al., 2012; Braden et al., 2017; Holter et al., 2019).

The literature linking memory, aging, and the cholinergic system led us to evaluate age-related differences in cholinergic activity after exposure to tasks requiring different memory types and memory demands. ChAT activity assays were performed in the dorsal hippocampus, ventral hippocampus, frontal cortex, perirhinal cortex, and entorhinal cortex, and these values were correlated with cognitive functioning. Aged female rats exhibited greater ChAT activity in the ventral hippocampus and frontal cortex compared to young rats, while age did not impact ChAT activity in the entorhinal cortex, perirhinal cortex, or dorsal hippocampus. These findings contribute to a larger body of literature studying aging in rodents, non-human primates, and humans, showing degradation of cholinergic functioning with aging in brain regions critical to learning and memory (Rinne, 1987; Araujo et al., 1990; Tayebati et al., 2002; Haley et al., 2011). Notably, much of this prior work was conducted in behaviorally naïve subjects. Behavioral testing itself is likely to significantly impact cholinergic outcomes, as this has previously been shown for neurotrophins (Bimonte et al., 2003), and studies have shown that the cholinergic system can be modified with food reward (Aitta-Aho et al., 2017), as well as with testing on a spatial discrimination task (Toumane et al., 1988). Assessing how complex behavioral testing impacts cholinergic function in specific brain areas amongst the broader system could add new dimensions and interpretations to age-related questions. For example, ChAT activity in the basal forebrain was a strong predictor of age-related spatial learning deficits as tested on the reference memory version of the Morris Maze in rats (Gallagher et al., 1990). Recent research has shown that cholinergic inputs modulate cue detection, cue salience, and top-down regulation of how cues are used to influence behavior (for reviews: Parikh and Bangasser, 2020; Venkatesan et al., 2020). These attentional processes are strongly linked to performance on memory tasks, as there is significant overlap between brain regions involved in both attention and memory (see review: Chun and Turk-Browne, 2007). In solving complex spatial navigation tasks, rodents typically rely heavily upon the presence of extramaze cues to locate the hidden platform(s); cholinergic output in related brain areas is therefore likely affected by this attentional demand. Hence, the observed increases in ChAT activity in both the frontal cortex and ventral hippocampus in aged female rats may be compensatory in nature, reflecting the brain's attempt to compensate for weaknesses elsewhere by ramping up cholinergic activity to ultimately yield improved attentional and memory outcomes.

The current findings suggest that with aging, when navigating a complex spatial maze task, cholinergic activity increases in brain regions important for spatial learning and memory outcomes; however, these increases in activity are insufficient to alter behavioral outcomes, as aged rats still demonstrated repeated learning and memory failures. These findings are in accordance with work from human studies (Dumas and Newhouse, 2011). Indeed, increases in prefrontal and parietal activity have been observed *via* functional MRI during evaluation on working memory and visual attention tasks in aged adults relative to young adults (Cabeza et al., 2004). This increase in brain activity with aging in regions critical to working memory processing is thought to be in response to a sufficient challenge; in fact, optimal activity within these brain regions to maximize working memory performance is shifted higher in aged adults than young adults evaluated *via* MRI (Rypma and D'Esposito, 2000). Furthermore, postmenopausal women administered the antimuscarinic drug scopolamine, known to impair working memory, showed increased MRI frontal lobe and hippocampal activity relative to placebo (Dumas et al., 2011). These complimentary findings shown in both the clinical and preclinical literature are further supported by the data shown herein. Specifically, for aged rats tested in the solely working memory WRAM, there was a positive correlation between ventral hippocampus ChAT activity and WMC errors, with greater cholinergic activity associated with increased working memory errors. This could be predicted in a compromised system, which ramps up cholinergic activity to compensate for age-related memory deficits. This is consistent with the assertion that “aging acts as an intervening variable” (Sarter and Bruno, 1998) in experiments designed to evaluate the vulnerability and restorative capacity of basal forebrain cholinergic projections, at least in the context of injury and degenerative processes. Conversely, for young rats, the relationship between cholinergic activity and behavioral outcomes is inverted: in a system that is not excessively challenged by the attentional demands of a spatial working memory task, greater cholinergic activity is sufficient to drive improvements in performance. This was reflected in the correlation for young rats whereby greater cholinergic activity in frontal cortex was associated with fewer working memory errors, thereby reflecting that increased cholinergic functioning is associated with better working memory outcomes in young adulthood. These findings demonstrate that, with aging, cholinergic modulation may be insufficient to reverse deficits in learning and memory processing, as aged rats remained impaired across the task; this fits with the broader understanding in the field that, with aging, the brain undergoes a multitude of changes beyond that of cholinergic dysregulation (Koebele and Bimonte-Nelson, 2017). The current work underscores that aging is a complex biological process, where behavioral outcomes are driven by a wealth of neurobiological changes. Future work should continue to evaluate the role that attentional demand, in addition to working memory and reference memory demand, plays in altering cholinergic outcomes across the female aging trajectory in both the clinical and preclinical domains.

## Conclusion

Taken together, the current findings support the precept that young rats are more capable of performing spatial working memory tasks compared to aged rats, and that the addition of a reference memory component yields a decrease in working memory performance with an increasing working memory load, such that young rats perform similarly to their aged counterparts. This interference of reference memory items on the working memory system suggests that the two are not orthogonal as once believed, and should be considered carefully when interpreting data from instruments that are often used to measure both working and reference memory constructs simultaneously, vs. tasks that measure them individually. Analyses of ChAT activity in both the ventral hippocampus and frontal cortex revealed an age effect, whereby aged rats had greater ChAT activity than young rats. The rigorous behavioral testing on a spatial learning and memory task likely impacted this outcome *via* a compensatory mechanism, whereby cholinergic output is increased in aged rats to aid in the attentional and mnemonic processing necessary to perform this complex spatial navigation task. Correlations demonstrate relationships between working memory and cholinergic activity within distinct brain regions across the two age groups, specific to the purely working memory task. This study highlights the complex relationships between age, cognitive demand, and cholinergic activity in discrete brain regions. Exploring relationships between working and reference memory can provide innovative breadth and depth into study outcomes, supplementing interpretations of better or worse performance including interactions between memory systems and attentional demands. The evaluation of not only working memory and reference memory outcomes in isolation, but of working and reference memory outcomes simultaneously and their relationships to one another, can give us the translatability necessary to better understand the complexities of age and neurodegenerative-related memory failure. This is critical, as in everyday life, people engage in activities that demand multiple cognitive domains simultaneously, including reference memory and working memory. Deciphering varied neurobiological profiles, as related to the ability to handle multidimensional memory demands and challenges, can optimize therapeutics for targeted functional outcomes in populations where cognition is impacted.

## Data Availability Statement

The datasets presented in this article are not readily available because the datasets from this study will not be made publicly available. Requests to access the datasets should be directed to Heather A. Bimonte-Nelson, bimonte.nelson@asu.edu.

## Ethics Statement

The animal study was reviewed and approved by the Arizona State University (ASU) Institutional Animal Care and Use Committee (IACUC).

## Author Contributions

VB completed all data analyses, generation of figures, and writing of manuscript. RH oversaw completion of project, with assistance of AC. MP and AC conducted behavioral evaluations. ZK conducted cholinergic tissue analyses. RG mentored ZK through tissue analysis and provided intellectual contributions to cholinergic sections. HB-N oversaw study design, data analysis, and manuscript completion. All authors contributed to the article and approved the submitted version.

## Conflict of Interest

The authors declare that the research was conducted in the absence of any commercial or financial relationships that could be construed as a potential conflict of interest.
